# Clinical Cases

**Published:** 1860-11

**Authors:** 


### CLINICAL CASES.

BY THE EDITOR.

Case 1. *Hemoptysis,—Premonition, &c.*—Mrs. D———, an intelligent American woman, aged about 24 years; was attacked with pulmonary hemorrhage on the 25th of August, 1860. Two days previously to the attack, she told her husband that she was about to have hemorrhage from the lungs, from which she should never recover, and became much depressed and nervous.

The husband, who was an educated physician, endeavored by reasoning and cheerful conversation to remove her fears, more especially as she was in her usual health. The impression, however, remained indelibly fixed on her mind, until the bleeding actually commenced.

I was called to see her a few hours after the flow of blood commenced. Found her face pale; her mind and nervous system much agitated; pulse 130 per minute, and moderately full; respiration short and hurried; with almost constant, though not rapid, expectoration of fresh blood. There was a great sense of oppression across the chest; or a mixed feeling of faintness and suffocation. At the time of my arrival, she had taken a solution of common salt as freely as her stomach would bear, and also a solution of gallic acid. Subsequently she took in succession, acetate of lead with morphine to allay the nervous excitement, alum, tinct. matico, tinct. gelseminum, oil turpentine, and quinine. But none of these, aided by the most perfect quietude, produced more than a very temporary and partial suspension of hemorrhage. So constant and considerable was the discharge of blood, that at the end of the third day, she was extremely pale; pulse frequent and soft; skin cool; with great sense of oppression across the chest, faintness, and frequent palpitations of the heart. The prostration was so great, and the bleeding so persistent, as to render the prognosis decidedly unfavorable.

At this stage of the case we advised the tinct. ferri murias, and tinct. of ergot, equal parts, of which 40 drops were taken every two hours. Under the use of this mixture the hemorrhage ceased in less than twenty-four hours, and has not been renewed since. The remedy was continued in smaller doses and at longer intervals for eight or ten days. There was no cough before the attack of hemorrhage, and there has been little or none since, although several weeks after the attack, there was sufficient alteration of the respiratory murmur and increased dulness on percussion, over the infra-clavicular region of one side, to justify the suspicion that turbercular deposit existed. There is evidently a hemorrhagic diathesis in the family, inasmuch as the father of the patient and one sister had previously died from Hemoptysis.

Case 2. *Hemoptysis in an Infant aged eight months.—Probable congenital tuberculosis.*—The child of Mr. B—, was a male, apparently healthy at birth: That is, to all outward appearance, it was well formed, plump in flesh, and active. It was soon observed, however, to breathe habitually short and more hurridely than natural. As it became old enough to make muscular efforts, or attempts at play, this peculiarity was more observable, and when a sleep, there was often a peculiar grunt with each respiration. The pulse was more frequent than normal, and there seemed to be an unusual susceptibility to cold, with occasional short cough. Still the child continued to be well nourished, and presented the outward aspect of fair health. At about the age of eight months, it was suddenly attacked with hemorrhage from the lungs. It was soon checked ; but within twenty-four hours, it returned, and so copiously as to produce suffocation almost immediately. A *post-mortem* examination revealed the existence of a single mass of tubercular deposit full an inch in diameter, located near the centre of the upper lobe of the right lung. The central part of this mass was softened to a semi-fluid consistence, while the circumference remained firm. The hemorrhage proceeded from the rupture of a small vessel on the margin of this tubercular mass. Two or three small masses of tubercle existed in the same lobe of the lung, but the rest of the lungs and all the other viscera were healthy. Both the parents appear to enjoy fair health, and there is no known hereditary tuberculous tendency in the family of either.

